# Identifying Risk Factors Affecting the Usage of Digital and Social Media: A Preliminary Qualitative Study in the Dental Profession and Dental Education

**DOI:** 10.3390/dj9050053

**Published:** 2021-05-08

**Authors:** Rayan Sharka, Jonathan P. San Diego, Melanie Nasseripour, Avijit Banerjee

**Affiliations:** 1 Centre for Dental Education, Faculty of Dentistry, Oral & Craniofacial Sciences, King’s College London, London SE1 9RT, UK; melanie.nasseripour@kcl.ac.uk; 2 Oral and Maxillofacial Department and Diagnostic Sciences, Faculty of Dentistry, Umm Al-Qura University, Makkah 24381, Saudi Arabia; 3 Centre of Oral Clinical Translational Sciences, Faculty of Dentistry, Oral & Craniofacial Sciences, King’s College London/Guy’s & St. Thomas’ NHS Hospitals Trust, London SE1 9RT, UK; avijit.banerjee@kcl.ac.uk

**Keywords:** undergraduate dental education, postgraduate dental education, continuing education, professionalism in dentistry, online education, digital media, social media, perceived risks

## Abstract

Aims: This study aimed to identify the risk factors of using DSM to provide an insight into the inherent implications this has on dental professionals in practice and trainee professionals’ education. Materials and methods: Twenty-one participants (10 dental professionals and 11 undergraduate and postgraduate dental students) participated in this qualitative study using semi-structured interviews in a dental school in the UK. The interviews were analysed and categorised into themes, some of which were identified from previous literature (e.g., privacy and psychological risks) and others emerged from the data (e.g., deceptive and misleading information). Results: The thematic analysis of interview transcripts identified nine perceived risk themes. Three themes were associated with the use of DSM in the general context, and six themes were related to the use of DSM in professional and education context. Conclusions: This study provided evidence to understand the risk factors of using DSM in dental education and the profession, but the magnitude of these risks on the uptake and usefulness of DSM needs to be assessed.

## 1. Introduction

The digital and social media (DSM) are defined in this study as internet-based applications that allow the creation and exchange of user-generated content and the associated digital technologies used in this creation and exchange [1]. Prominent examples of DSM include a range of communication modes across social media platforms (e.g., Twitter, Facebook, and Instagram), instant messaging apps (e.g., WhatsApp, WeChat), and video conferencing platforms (e.g., Zoom, MS Teams) through users’ smartphone or computer devices. The current 3.8 billion global DSM users suggest there is an increasing impact of DSM in the private and working lives of the general population [2]. This has extended to dental students and dental professionals, with up to 98% of dental students and 75% of dental professionals reporting that they used DSM for professional and personal use [3,4], for example, for communication (with friends and family), professional education (teaching and learning dentistry), or dental practice advertisement (promoting oral health care and marketing dental services) [5,6,7]. The literature suggests that both user groups have highlighted perceived risks associated with the use of DSM [8,9]. Perceived risks can be defined as a possible loss, harm, or uncertainty of using DSM services [10]. Previous studies in the general online services context have applied this definition to understand and address the risk associated with the use of information technology and social media to advance the uptake of its services in the general population [10,11]. In this paper, these are referred to risks associated with the use of DSM in the general context.

Dental professionals in academic and practice settings have a professional reputation as well as regulatory requirements to uphold and be sensitive to since they have a professional standing that elicits society’s trust [12]. Concerns about breaching patients’ confidentiality and compliance with regulations and professional standards could also hinder the uptake of DSM in the professional context [13]. In this paper, these are referred to risks associated with the use of DSM in the professional and education context.

Despite the ongoing debate of the usefulness of DSM in dentistry [13], there have been few attempts to identify the risks of using DSM between the contexts in terms of general usage and how usage specific to dental education and profession affects the uptake of using DSM. Additionally, although this body of knowledge about dental students and professionals’ compliance is crucial [14,15], there are several other general and professional risks that need to be explored and discussed. Moreover, without a doubt, the COVID-19 pandemic has brought immense challenges to dental education worldwide, which has caused a sudden transition to implement online technologies, including DSM [16]. Therefore, identifying risk perceptions associated with the implementation of such technologies seems to be justified and rational. This study aimed to identify the risk factors of using DSM to provide an insight into the inherent implications this has on dental professionals in practice and the education of trainee professionals. Dental students and professionals’ risk perceptions appeared to differ from the public risk perceptions of using DSM. The research questions were:

RQ1: What are the perceived risks that can be identified associated with dental students and professionals using DSM in dental education and practice?

RQ2: Do the identified risks associate specifically with dental education and professional context and/or general usage context?

## 2. Materials and Methods

### 2.1. Participants and the Setting

Ethical approval was granted by King’s College London Ethics Committee (LRS-18/19-8867). The study participants included dental students (undergraduate/postgraduate students) and dental professionals (clinical teachers providing dental care and teaching) and were recruited by email and invitation posters using purposive sampling to give participants a chance to participate in this study voluntarily [17,18]. The interviews were conducted until the interview responses reached saturation, that is when the responses did not provide new perceived risk themes during interview analysis [19]. Using these procedures, a total of 21 participants, 11 dental students, and 10 dental professionals participated in this study, of which, 12 were male, and nine were female. The median age of the dental students was 22 years (range 18–35), and for the dental professionals, the median age was 33 years (range 30–51).

### 2.2. Interview Procedure

A semi-structured interview guide, including open-ended questions followed by prompting questions to probe into details, was followed during interviews to elicit responses about perceived risks. The main aspects discussed in the interviews included the anticipated perceived risks and factors that influence their current use and activities of DSM and any particular challenges that could be hindering dental students and professionals’ use of DSM in general and professional contexts. The interview guide was piloted with two dental students to ensure that the questions were appropriate and understandable [20]. The interviews were conducted face-to-face in a quiet office over five months between 2019 and 2020 and lasted between 15–30 min. They were audio-recorded and transcribed verbatim. An incentive Amazon voucher worth £10 was offered to each participant at the end of the interview in appreciation of their time.

### 2.3. Thematic Analysis Process and Coding

The initial code descriptions were developed and informed by previous studies [3,4,5,6,7,8,9,10,11]. Following the transcription of each interview, the main researcher read the transcripts to highlight and note the risks identified in the process of familiarisation. To ensure systematic coding and reflexivity, phrases were first coded into perceived risks and other non-risks, the risk phrases were then scrutinised to produce preliminary codes of perceived risks, which were peer-validated with a second researcher (J.S.D.) to ensure the rigour and validation of the codes. Coding descriptions not identified previously in the literature which emerged from the data were added to the coding scheme [21]. This process formed a working coding scheme which was subject to several iterations of refinement to review and refine each risk code (Table 1). To ensure the reliability of a coding scheme, the assessment of the inter-coder reliability (ICR) and the level of consistency was established following the methodology of MacPhail et al. (the overall Kappa score = 0.70) [22]. A consensus agreement was achieved through the feedback from the two coders, with any discrepancies between the two coders reconciled.

**Figure 1:**
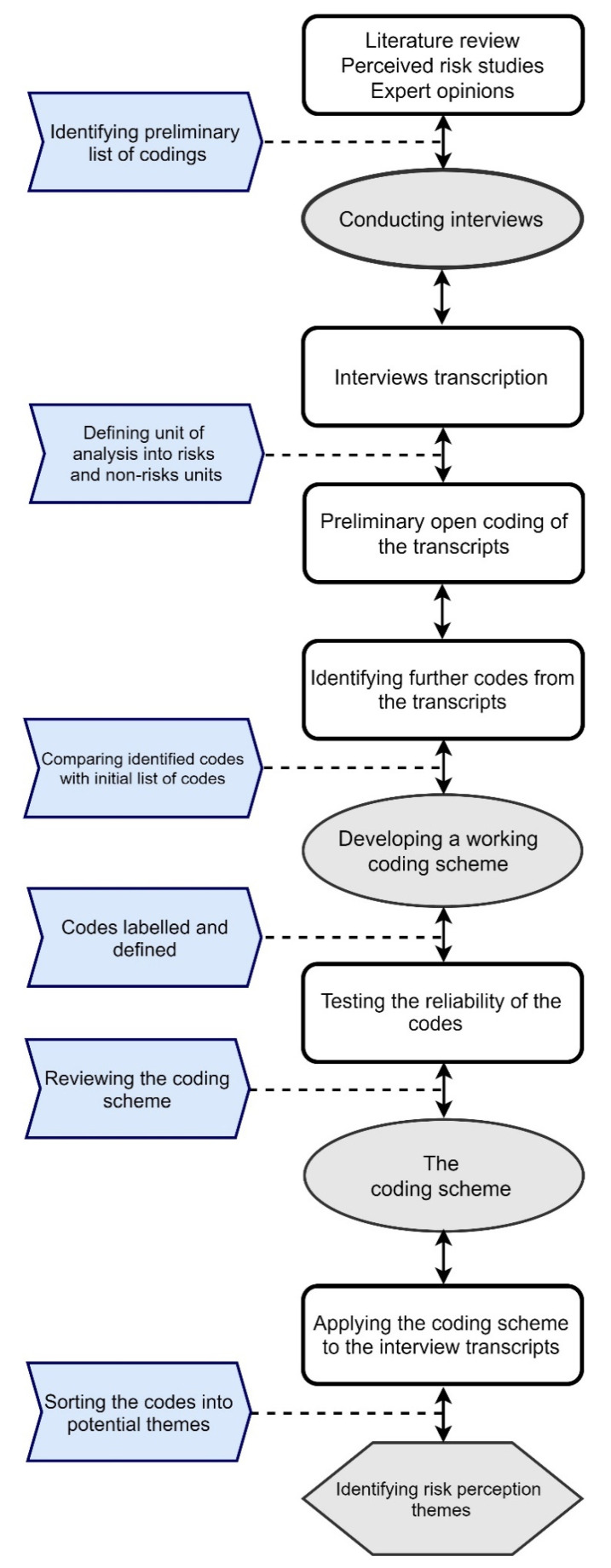
Process of analysis and developing themes.

All transcripts were then imported into Nvivo12 software (QSR International Pty Ltd., Melbourne, Australia) for thematic analysis, and each code of the coding scheme was entered as a node. The illustrative phrases of perceived risks from all interview transcripts were assigned into each code. The researchers then examined the coded data for further validation. The process of coding and developing risk codes is summarised in Figure 1.

## 3. Results

**Figure 2:**
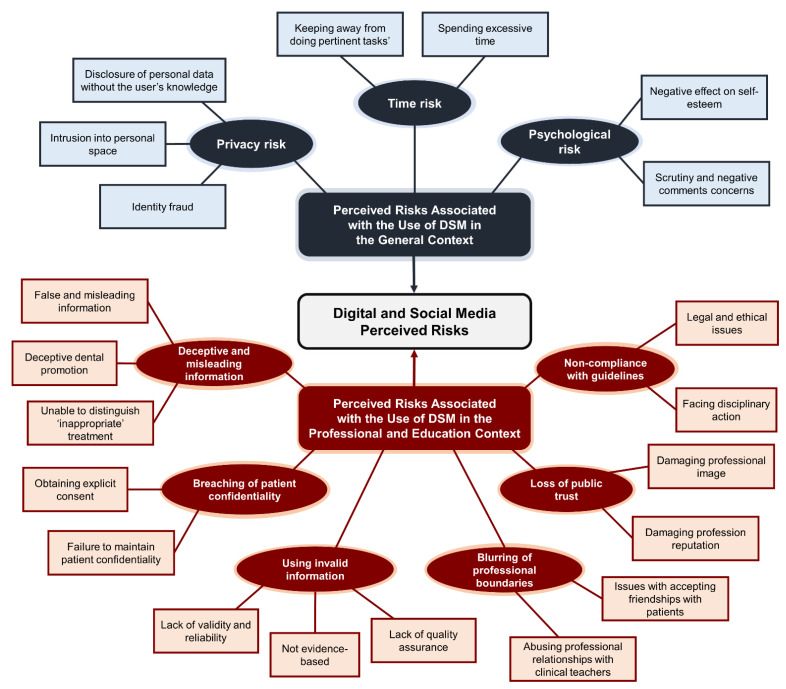
Thematic map showing the 21 sub-themes (rectangular shape) and nine main themes (oval shape).

From the thematic analysis of the 21 interviews, a total of 302 phrases were identified and coded (140 risk codes and 162 non-risk codes). The 140 risk codes were assigned to 21 sub-themes, then clustered to identify nine main risk themes (Figure 2), three of which were associated with using DSM in the general context, and six themes which were associated with using DSM in professional and education context. In the following Section 3.1 and Section 3.2, each perceived risk theme is defined and presented with illustrated exemplar quotes. Table 2 presents the distribution of themes by the number of participants and the frequency of occurrence.

### 3.1. Perceived Risk Themes Associated with the Use of DSM in the General Context

#### 3.1.1. Theme (1): Privacy Risk

This theme showed highest occurrence (25 codes) across interviews, with seven out of 11 dental students having concerns about revealing personal information, such as posting and sharing private photos and specific personal life areas to the public on DSM. They preferred to use DSM within a narrow circle of people, such as families and friends, without intrusion from stranger users. For example, one dental student mentioned that “My personal accounts are private, and only my friends and family members follow me” (DS.9).

Furthermore, seven dental professionals considered that the wide availability and easy accessibility of personal information on DSM could lead to identity fraud and ID theft. For example, one dental professional described how DSM allows scammers to steal users’ identity and personal information, leading to damage to a person’s credit or reputation. He explained, “someone can create a fake account and post stuff as yourself, especially if you are a well-known person; you definitely have friends and enemies, and they can use your information on DSM and write untrue information about you” (DP.6).

#### 3.1.2. Theme (2): Psychological Risk

Seven out of 11 dental students reported that DSM is an open space for users to share and post contents. This kind of usage is not protected from scrutiny and negative comments, which adversely affect feelings and self-esteem. For example, one dental student described this situation by saying that “some people posting their work, they think it is good but everyone else making fun of their work saying that your work is bad, it is too invasive; there are always risks like humiliation, and personally I do not like that” (DS.9). Similarly, seven dental professionals mentioned that they decreased their interaction with people through DSM due to the possibility of receiving negative remarks from others. One dental professional commented “I do not like to use DSM to avoid any unnecessary negative comments that make me inconvenient” (DP.5).

#### 3.1.3. Theme (3): Time Risk

A concern of spending excessive time on DSM was discussed during interviews. Eleven out of 21 of dental students and professionals had concerns of spending too much time on DSM. One dental student noted that DSM platforms use a variety of means that encourage users to engage and spend time. He explained that “you can spend ages browsing and achieving nothing in your work, especially if you have a deadline” (DS. 11).

Also, five dental professionals described the same issue, supporting this opinion, and reported that using DSM in browsing and socialising with others wastes time and hinders them from doing pertinent tasks. “With regards to personal use, it is very time-consuming; it keeps you away from daily physical activities just by staying on it and browsing” (DP. 8).

### 3.2. Perceived Risk Themes Associated with the Use of DSM in the Professional and Education Context

#### 3.2.1. Theme (4): Using Invalid Information

This theme presented the second highest occurrence across interviews (20 codes), with seven dental students perceiving uncertainty about using information shared on DSM because the quality of the information was different from that taught in the authentic academic environment. One dental student noted that “there is much information out there so that you do not know what is evidence-based and what is not” (DS.4). Another student supported this claim by saying that “sometimes it is questionable, and sometimes the stuff we learnt is different from what they are doing” (DS.6).

Similarly, seven dental professionals acknowledged that the main drawback of using DSM for learning is a lack of assurance of the quality of the information; “there is no filtering feature that helps the user to distinguish between the right and wrong information” (DP.9), explained one dental professional.

#### 3.2.2. Theme (5): Non-Compliance with Guidelines

Dental students and professionals were aware of DSM guidelines issued by professional governing bodies and subsequent disciplinary actions. Six dental students reported their cautious use of DSM to avoid violation of these guidelines. For example, one student noted that “if you are a dental student, you have to be aware and do not make a mistake” (DS.11). Additionally, four dental professionals mentioned the importance of complying with authority guidelines. One dental professional noted, “it is good to make sure that all the regulations are followed” (DP.3).

#### 3.2.3. Theme (6): Breaches of Patients’ Confidentiality

Five dental students stated that, even with patient consent, using DSM to post or share patient’s information still presents issues as “all photos are on the internet technically forever so any person can download and share it. There is a sticky situation in doing that” (DS.9) one dental student explained. Likewise, five dental professionals disagreed with using DSM to share dental cases. One dental professional said, “I kind of see it like a breach of patient confidentiality, even with the obtained consent form” (DP.6).

#### 3.2.4. Theme (7): Deceptive and Misleading Information

Five dental students mentioned that DSM could be used to share information which misled or showed inaccurate dental treatments or promotions. “The public, unfortunately, gets a lot of wrong information; I think people are promoting information to get benefit from it such as [Snap-On Smile]” (DS.3), explained one dental student. Additionally, one student gave an example of such deceptive dental promotion on DSM. He stated that “You see cases posted on DSM such as [3 to 3 crowns or veneers]; did the patients need that? I think that’s where the danger is, when you go online, and people think this is the normal treatment” (DS.7).

Similarly, four dental professionals discussed other aspects of misinformation such as the dental contents shared on DSM for the public not being created by specialised health care. One professional explained that “you can easily get fake information or untrue information because not all the posts are created or written by dental professionals” (DP. 6).

#### 3.2.5. Theme (8): Blurring of Professional Boundaries

Thirty-eight percent of students and professionals interviewed felt that the line between personal and professional blurred when interacting on DSM. Six dental students described the type of interaction with dental professionals as inappropriate. One dental student said, “if I tag him on a Facebook post and say hi mate, this would be less professional” (DS.1). Similarly, dental professionals believed that their position as educators placed them in a conflicting position when interacting with their dental students on DSM. Therefore, they favoured maintaining a clear boundary between professional and personal lives. One dental professional noted, “I think it is good to have a boundary between professional life and personal life, and I think sometimes that barrier can be quite abused” (DP.3).

#### 3.2.6. Theme (9): Loss of Public Trust

Dental students reported that using DSM could reflect negatively on their reputation and affiliated institutions if they share unprofessional content. “I would not post anything which someone could question about me as a dental professional” (DS.8), explained one dental student. Similarly, two dental professionals stated the same perception. For example, one dental professional said, “if you set up your professional profile, you cannot be perceived like a normal user! You cannot be a person who is having a party on Sunday or the weekend and posting it on DSM. You have to be a professional person” (DP. 2).

## 4. Discussion

This study attempted to identify the risk perceptions specific to dental students and dental professionals’ usage of DSM and discuss how their perceptions are different from the public users. Interestingly, some perceived risks were consistent with the use of DSM in the general context identified in previous studies [10,11], including psychological, privacy, and time risks, and others were associated with the use of DSM in the professional and education context, such as non-compliance with guidelines, breaching patient’s confidentiality, and using invalid information [8,9,14,15].

The identified risk themes indicated that DSM impacted both the professional and personal life of dental students and professionals. When they used DSM in their personal life, they perceived similar risks as users perceived in a general e-services context [10,11]. However, the significant theme was privacy risks because the nature of DSM usage is to connect and interact with colleagues or friends by revealing information, such as sharing thoughts, photos, movements, facial expressions, and interests with others. This type of activity is neither guarded against strangers nor effectively able to be protected. Such concern has been found to be an essential factor affecting the unwillingness and dissatisfaction to use DSM [11,23]. A comparable outcome was reached previously among a sample of dental students and dental educators, when 60% of them stated that privacy concerns were the major reason for not using DSM [4,15]. Nevertheless, in this study, dental students and professionals extended their perceptions to include other aspects of privacy issues, such as a high incidence of hacking and personal data breaches, making them speculate the security of their personal information on DSM.

Interestingly, psychological issues emerged from the interviews, which is in line with a growing number of studies that suggest a possible association between using DSM and a potential negative influence on self-esteem, depression, and emotions [24,25]. In this study, this could be explained by the fact that most DSM interactions were unprotected from negative remarks that may affect dental students and professionals’ self-esteem, causing feelings of distress. Similarly, Davila et al. observed that depressive symptoms, including sadness and feelings of worthlessness, were associated with the quality of online communications and interactions they received while using DSM [26]. Another explanation could be due to the excessive time that dental students and professionals spend on DSM. According to a study by Iwamoto and Chun, significant positive correlations were found between the hours spent on DSM and stress, anxiety, and depression in a sample of undergraduate students [25].

Approximately half of the interviewees admitted that they were spending up to two hours per day on DSM, which could negatively impact students’ academic progress and professionals’ daily work. This theme indicated that DSM platforms provide various means that genuinely encourage users to allocate considerable time, ranging from connecting with friends to browsing the latest news and exploring other posts, making it time-consuming. These findings are supported by a previous study among dental students who believed that using DSM was time-consuming and distracted them from their studies [27].

With regards to risks associated with the use of DSM in professional and education context, the interviewees shed light on a crucial risk, that is, invalid information. They noted that DSM has become an open-learning resource, providing educational materials through a user-generated content feature; however, much information shared is neither checked for its validity nor based on a reliable source. This important finding has been discussed in the literature and could have serious implications for dental education [8]; hence, dental students and professionals need additional training and education to search for evidence-based information effectively. In a recent study, Khatoon et al. demonstrated that dental students might not be competent in the skills and experience required to scrutinise the information shared online [28]. With the current dramatic move to online education and the imminent surge in the adoption of digital technologies to disseminate educational contents due to the COVID-19 pandemic, such crucial training has become a necessity [29]. Furthermore, nearly half of the dental students and professionals stated that they could be in breach of patient confidentiality if they shared their work, including clinical cases, on DSM. Additionally, some of them reported that posting patients’ photos was entirely unacceptable, even if consent was obtained. The governing dental body, such as the General Dental Council (GDC) in the UK, declared that posting unidentifiable patients’ photos on social media was prohibited without explicit consent from patients [30]. Patients should manifest an understanding of this reality before consenting. Several studies showed differences in the perceptions of dental students relating to this argument, suggesting the importance of introducing e-professionalism education to the curriculum of undergraduate and postgraduate students to help them avoid these pitfalls when using DSM [3,14]. Furthermore, a point worth consideration is that dental students and professionals must be familiar with the dental school and hospital guidelines governing DSM use and strictly comply to them as the laws in different countries about posting patient images on DSM could differ.

Even though this study aimed to understand the risk perceptions that concern dental students and professionals themselves as health care providers, they reported a risk that affects public users, including their patients. DSM creates uncontrolled online spaces, housing communication and dental advertising that might contain a potential threat of misinformation, which could affect the delivery of treatment care or management of patient expectations of dental practice. Some dental professionals warned of such information [31], especially for patients among young groups who are very sensitive to the perception of their dental, facial, and body appearance [32]. Shuttleworth and Smith stated that the rising incidence of cases that are influenced by the misinformation posted on DSM could open up new challenges for stakeholders in the dental health system, with the increased sharing of commercially-directed advertisements on such uncontrolled platforms [33].

It is noteworthy to mention that the impact of the ample challenges brought by COVID-19 on the perceptions of dental students and professionals have not been investigated in this study [16]. Future studies to analyse the impact of COVID-19 on the use of DSM in the professional development and training of dental students are required.

Furthermore, it is imperative to utilise quantitative methodology, e.g., a questionnaire instrument with a larger number of participants, to generalise the identified risks and assess differences between groups and their magnitude on the uptake of DSM in dental education.

## 5. Conclusions

This study presented the critical risks of DSM that influence dental students and professionals’ usage of DSM that have not been previously explored in such a comprehensive approach. Furthermore, the combination of perceived risks in the developed framework as presented in Figure 2 is novel and provides a unique contribution to dental schools’ policymakers to widen their lens about risks that affect dental students and professionals, which are different from those previously identified among public users. Dental educators can benefit from the findings of this study, which could be adopted as an introductory guideline to advance the usage of DSM among dental students and professionals with the guidance of existing governing bodies regarding social media.

Finally, dental students and professionals need to be more vigilant in using DSM so as not to damage the image of the profession and lose public trust. They need training and reassurance on how to overcome these risks and acquire a clear sense of how they might open up new avenues for knowledge dissemination, promotion ideas, and connection with the public, including their patients.

## Figures and Tables

**Table 1:** The coding scheme developed based on literature review and interviews to code the interview transcripts.

Codes	Description	Example
Negative effect on self-esteem and self-image	The possibility of an adverse effect on the users’ peace of mind or self-esteem from using DSM.	“I believe that DSM does affect self-esteem due to exposing users to a vast number of photos, such as ideal body image, that would affect self-esteem or self-image”.
Scrutiny and negative comments concerns	The possibility of receiving negative comments and criticism from using DSM.	“I will receive negative remarks from others if I use DSM”.
Disclosure of personal data without the user’s knowledge	The potential loss of control over personal information leads to the information being used without the user’s knowledge or permission.	“Someone else can take the photo that I posted on my profile and use it without my consent”.
Intrusion into personal space	The state when personal/private life is observed or disturbed by others.	“Using DSM would allow others to observe my private life”.
Fear of hacking and identity fraud	The potential loss of control over the DSM profile and account due to hacking and criminal attacks.	“Internet hackers (criminals) might take control of my checking account if I used a DSM”.
Spending excessive time	The possibility of losing time when using DSM by wasting time searching and browsing different activities on DSM.	“There is a possible time loss due to engaging in different activities on DSM”.
Keeping away from doing pertinent tasks	The possibility of losing the time for doing important tasks (e.g., studying, exercising, etc.)	“If I had begun to use DSM, there are chances that I will lose time doing other essential tasks”.
Social loss	The potential loss of status in one’s social group due to adopting DSM.	“My signing up for and using DSM would lead to a social loss for me because my friends and relatives would think less highly of me”.
Financial loss	The potential loss of money due to adopting DSM.	“There are the chances that you stand to lose money if you use DSM”.
Performance issues	The possibility of the DSM not performing as it was designed and advertised.	“DSM might not perform well and create problems”.
Lack of validity and reliability of the information	The possibility of using/sharing unreliable and invalid information on DSM.	“The information posted on DSM is poorly referenced and unreliable”.
Not evidence-based information	The possibility of using/sharing not evidence-based information on DSM.	“On YouTube and Google, you have to be careful in terms of what you are taking as evidence-based or not”.
Information has a lack of quality assurance	The possibility that the information shared on DSM is not critically appraised to ensure its quality.	“The information posted on DSM has a lack of quality assurance.”
Facing disciplinary action from regulatory bodies	The possibility of facing disciplinary action from using DSM.	“You must be aware and not make a mistake when using DSM to avoid disciplinary action”.
Legal and ethical issues	The possibility of exposure to legal penalties from using DSM.	“There is a risk of legal and ethical issues associated with DSM usage”.
Damage the professional image and reputation	The state of damage the professional image when using DSM inappropriately.	“I think using DSM is risky because anything you put could stay forever and affect your professional image”.
Not following governing bodies guidelines	The possibility of violating guidelines when using DSM.	“It is crucial to make sure that all the regulations are followed when using DSM”.
Breaching patient confidentiality	The possibility of violating and breaching patients’ confidentiality when using DSM.	“I believe that using DSM is good as long as they do not expose patient privacy”.
Obtaining explicit consent	The possibility of sharing patients’ information on DSM without explicit consent.	“There is a risk of sharing patients’ photos on DSM without explicit consent”.
False and misleading information	The state of sharing misleading information when using DSM.	“There is lots of misleading and false information shared on DSM”.
Being deceptive in dental promotions	The state of sharing deceptive dental promotions on DSM.	“Someone can easily photoshop and play with the quality of clinical work and enhance how the treatment looks”.

**Table 2:** Distribution of themes, by number of total participants (*n* = 21) and number of total occurrences (*n* = 140).

Perceived Risk Themes Associated with the Use of DSM in the General Context	Number of Participants	Number of Occurrences
1. Privacy risks	14	25
2. Psychological risks	14	18
3. Time risks	11	13
**Perceived Risk Themes Associated with the Use of DSM in the Professional and Education Context**	**Number of Participants**	**Number of Occurrences**
4. Using invalid information	14	20
5. Non-compliance with guidelines	10	17
6. Breaches of patients’ confidentiality	10	15
7. Deceptive and misleading information	9	12
8. Blurring of professional boundaries	8	10
9. Loss of public trust	7	10

## Data Availability

The data presented in this study are stored in a digital secured place of King’s College London and available upon reasonable request.
